# Supplementary material for: Structure/Activity Analysis of TASK-3 Channel Antagonists Based on a 5,6,7,8 tetrahydropyrido[4,3-d]pyrimidine
Source: Int J Mol Sci. 2019 May 7;20(9):2252. doi: 10.3390/ijms20092252 (PMC6539479; doi:10.3390/ijms20092252)
Supplement: Supplementary file 1 [file ijms-20-02252-s001.pdf]

Structure/activity analysis of TASK-3 channel antagonists based on a 5,6,7,8 tetrahydropyrido[4,3-d]pyrimidine.

David Ramírez, Mauricio Bedoya, Aytug K. Kiper, Susanne Rinné, Samuel Morales-Navarro, Erix W. Hernández-Rodríguez, Francisco V. Sepúlveda, Niels Decher and Wendy González.

Table S1. Residues in the Fenestration (F) and the Pore during the MDs[a]

Residue	T3twiOO	T3tre2OO	T3tre1CC
L122	F P	F P	P
Q126	P	P	P
G231	NP	NP	NP
G236	F P	F P	P
A237	NP	NP	NP
L239	F P	F P	P
L244	P	P	P
L247	P	P	P
T248	P	P	P

P: Pore; **F**: Fenestration; **NP**: No presence

[a] A residue is considered as part of a cavity if it remains more than 5 ns in the cavity.

Table S2. Structure and biological activity data for 5,6,7,8-tetrahydropyrido- [4,3-d]pyrimidine (THPP) analogues [1].

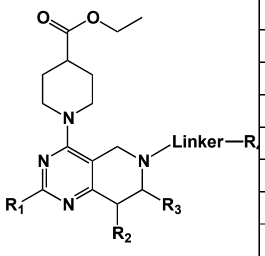

Compound	Linker	R_1	R_2	R_3	R_4	IC_{50} (µM)
9c	CH_2	H	H,H	H,H	Ph	0.71 ± 0.08
10b	CONH	H	H,H	H,H	CH_2Ph	3.4 ± 0.4
10c	CONH	H	H,H	H,H	p-biphenyl	9.6 ± 0.5
11a	SO_2	H	H,H	H,H	CH_2Ph	3.6 ± 0.4
11b	SO_2	H	H,H	H,H	4-Cl-Ph	3.9 ± 0.5
11c	SO_2	H	H,H	H,H	3-Cl-Ph	0.7 ± 0.07
11d	SO_2	H	H,H	H,H	2-Cl-Ph	0.9 ± 0.1
12a	CO	H	H,H	H,H	Ph	3.6 ± 0.77
12b	CO	H	H,H	H,H	4-MeO-Ph	1.6 ± 0.18
12c	CO	H	H,H	H,H	4-Br-Ph	0.43 ± 0.06
12d	CO	H	H,H	H,H	4-Me-Ph	0.31 ± 0.08
12e	CO	H	H,H	H,H	4-*c*Hx-Ph	0.12 ± 0.01
12f	CO	H	H,H	H,H	4-Ph-Ph	0.074 ± 0.009
13a	CO	H	Me,Me	H,H	4-Ph-Ph	0.57 ± 0.03
13b	CO	H	H,H	Me,Me	4-Ph-Ph	0.65 ± 0.03
14b	CO	Me	H,H	H,H	4-Ph-Ph	0.26 ± 0.05

Compound	R_1R_2	IC_{50} (µM)
17a	$-CH_2CH(OCH_3)CH_2-$	15 ± 6

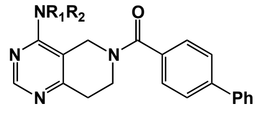	17b	–$CH_2CH_2CH_2CH_2$–	27 ± 2
	17c	–$CH_2CH_2CH_2CH_2CH_2$–	4 ± 1.1
	17d	–$CH_2CH_2OCH_2CH_2$–	4.6 ± 1.1
	17e	–$CH_2CH_2SO_2CH_2CH_2$–	0.57 ± 0.06

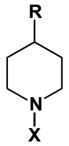 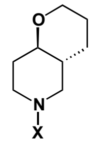 18, 19, 21-24 20 X 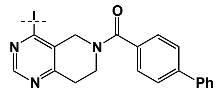	Compound	R_1	IC_{50} (μM)
	18	SO_2Me	0.082 ± 0.005
	19	OMe	0.135 ± 0.037
	20a	*cys*	0.45 ± 0.02
	20b	*trans*	0.07 ± 0.007
	21	$C(OH)Me_2$	0.05 ± 0.006
	22	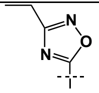	0.07 ± 0.01
	23	$C(O)CH_2CH_2CH_3$	0.035 ± 0.005
	24	$C(O)CH_2CH_3$	0.08 ± 0.009

Table S3. Reported activity and calculated affinities of compounds of THPP series in the different TASK-3 homology models.

THPP series	IC_{50} (μM)	pIC_{50} (μM) Ln (100/IC_{50})	MM-GBSA ΔG_{Bind} (kcal/mol)		
			T3tre1CC	T3twiOO	T3tre2OO
PK-THPP (23)	0.035	3.46	-81.53	-115.84	-89.88
21	0.05	3.30	-72.08	-88.46	-80.49
20b	0.07	3.15	-75.68	-95.64	-82.68
22	0.07	3.15	-77.37	-92.57	-84.26
12f	0.074	3.13	-71.65	-89.14	-82.16
24	0.08	3.10	-75.80	-88.37	-84.90
18	0.082	3.09	-68.64	-87.17	-84.48
12e	0.12	2.92	-73.04	-84.48	-83.60
19	0.135	2.87	-69.93	-78.83	-80.96
14b	0.26	2.59	-66.77	-78.51	-80.94
12d	0.31	2.51	-66.28	-76.44	-74.26
12c	0.43	2.37	-68.53	-73.37	-75.23
20a	0.45	2.35	-71.83	-86.20	-76.53
13a	0.57	2.24	-74.24	-73.42	-75.47
17e	0.57	2.24	-74.41	-75.15	-77.15
13b	0.65	2.19	-73.51	-69.76	-74.13
11c	0.7	2.15	-70.97	-72.49	-81.53
9c	0.71	2.15	-73.37	-76.17	-80.03
11d	0.9	2.05	-72.22	-72.09	-78.14
12b	1.6	1.80	-72.25	-67.80	-77.58
10b	3.4	1.47	-70.42	-72.14	-74.77

11a	3.6	1.44	-71.63	-68.79	-76.16
12a	3.6	1.44	-67.74	-66.78	-78.49
11b	3.9	1.41	-67.89	-64.34	-71.37
17c	4	1.40	-60.84	-60.01	-71.38
17d	4.6	1.34	-69.47	-61.94	-73.85
10c	9.6	1.02	-61.73	-75.85	-66.66
17a	15	0.82	-57.84	-58.35	-62.58
17b	27	0.57	-55.76	-69.63	-59.21

Table S4. Interactions of compounds of THPP series with TASK-3. Summary of the interactions of compounds of THPP series (17b, 20b, 21, 22 and 23) with the `hits´ and the threonines of the selectivity filter of TASK-3. Interactions between the ligands and the protein were determined using the "Ligand interaction diagram" tool of the Schrödinger suite (Maestro, Schrödinger, LLC, New York, NY, 2017).

#	Lig name	Protein residue	Interaction	Structure	Group (residue)	Distance (Å)
1	17b	L247 (Subunit B)	Hydrophobic	Biphenyl (C16 atom) 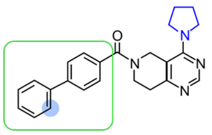	Delta methyl 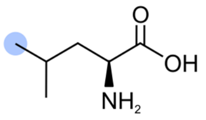	C-C 3.73
2	17b	L244 (Subunit B)	Hydrophobic	Biphenyl (C15 atom) 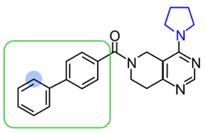	Gamma carbon 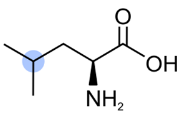	C-C 3.80
3	17b	T248 (Subunit B)	Polar	Biphenyl (H of C18 atom) 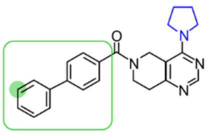	Nitrogen 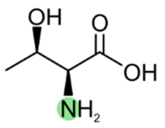	H-N 3.52
4	17b	L244 (Subunit A)	Hydrophobic	Biphenyl (C7 atom) 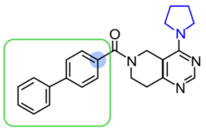	Delta methyl 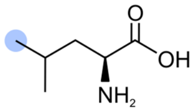	C-C 3.83
5	17b	Q126 (Subunit A)	Polar	Tetrahydropyridine (H of C6 atom)	Nitrogen of the amine	H-N 3.15

				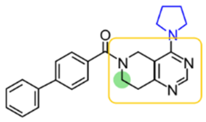	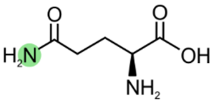	
6	17b	G236 (Subunit B)	Polar	H of pyrimidine carbon<br>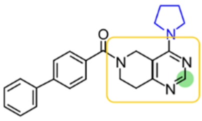	Oxygen<br>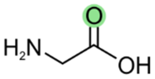	H-O<br>2.60
7	17b	L239 (Subunit B)	Hydrophobic	Pyrimidine nitrogen<br>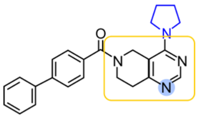	Backbone carbon<br>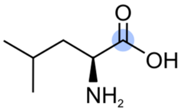	N-C<br>4.00
8	17b	L122 (Subunit B)	Hydrophobic	Substituent (C22 atom)<br>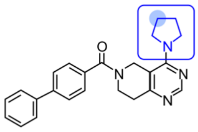	Delta methyl<br>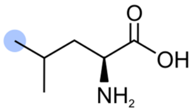	C-C<br>3.86
9	17b	Q126 (Subunit B)	Polar	Substituent (N atom)<br>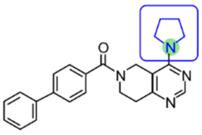	Hydrogen of the amine<br>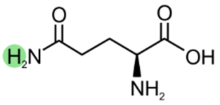	N-H<br>2.14
10	20b	L244 (Subunit A)	Hydrophobic	Biphenyl (C10 atom)<br>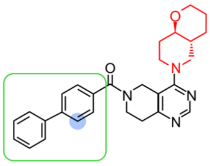	Delta methyl<br>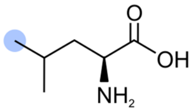	C-C<br>3.70
11	20b	L244 (Subunit B)	Hydrophobic	Biphenyl (C11 atom)	Delta methyl	C-C<br>3.89

				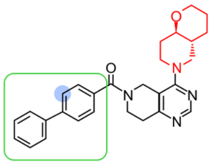	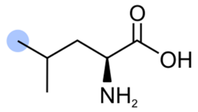	
12	20b	L247 (Subunit B)	Hydrophobic	Biphenyl (C15 atom)<br>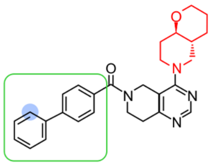	Delta methyl<br>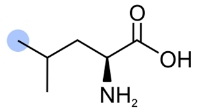	C-C 3.64
13	20b	T248 (Subunit B)	Polar	Biphenyl (H of C17 atom)<br>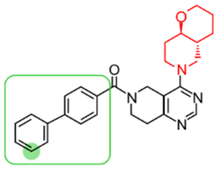	Nitrogen<br>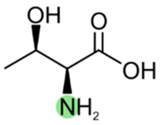	H-N 3.37
14	20b	Q126 (Subunit A)	Hydrogen bond	Carbonyl<br>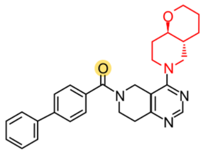	Hydrogen of the amine<br>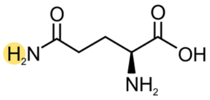	O-N 2.21
15	20b	L239 (Subunit A)	Hydrophobic	Pyrimidine nitrogen<br>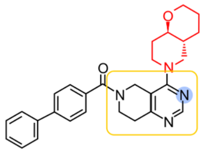	Delta methyl<br>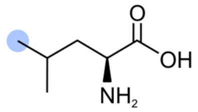	N-C 3.71
16	20b	Q126 (Subunit B)	Hydrogen bond	Pyrimidine nitrogen<br>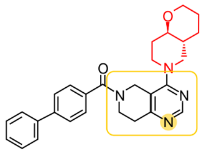	Hydrogen of the amine<br>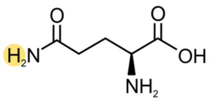	N-H 1.95

17	20b	G236 (Subunit A)	Polar	Substituent (H of C27 atom)<br>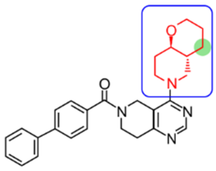	Oxygen<br>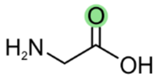	H-O 2.68
18	20b	L239 (Subunit A)	Hydrophobic	Substituent (C21 atom)<br>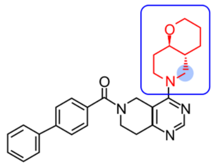	Delta methyl<br>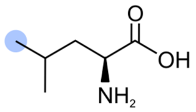	C-C 3.95
19	20b	L122 (Subunit A)	Hydrophobic	Substituent (C24 atom)<br>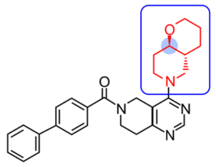	Delta methyl<br>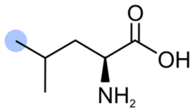	C-C 3.98
20	21	L247 (Subunit B)	Hydrophobic	Biphenyl (C17 atom)<br>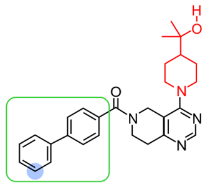	Delta methyl<br>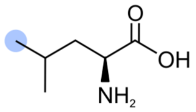	C-C 3.63
21	21	L244 (Subunit B)	Hydrophobic	Biphenyl (C18 atom)<br>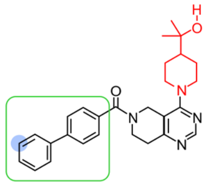	Beta carbon<br>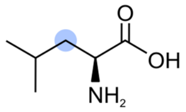	C-C 3.81
22	21	Q126 (Subunit A)	Hydrogen bond	Carbonyl<br>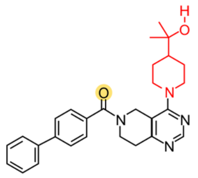	Hydrogen of the amine<br>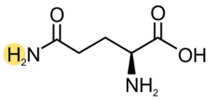	O-H 2.11
23	21	L122 (Subunit A)	Hydrophobic	Tetrahydropyridine (C4 atom)	Delta methyl	C-C 3.60

				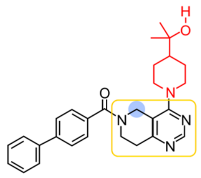	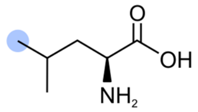	
24	21	L239 (Subunit B)	Hydrophobic	Pyrimidine carbon 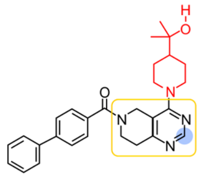	Delta methyl 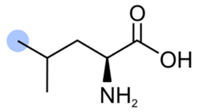	C-C 3.61
25	21	G236 (Subunit B)	Polar	Tetrahydropyridine (H of C5 atom) 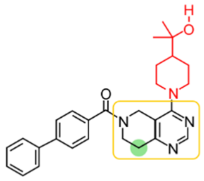	Oxygen 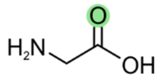	H-O 2.89
26	21	T93 (Subunit A)	Hydrogen bond	Hydroxyl 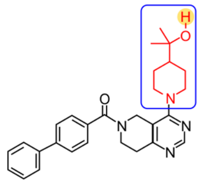	Gamma oxygen 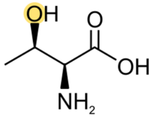	O-H 1.90
27	22	T248 (Subunit B)	Hydrophobic	Biphenyl (C18 atom) 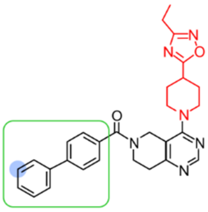	Gamma carbon 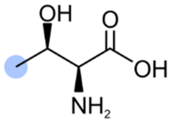	C-C 3.40
28	22	Q126 (Subunit A)	Hydrogen bond	Carbonyl 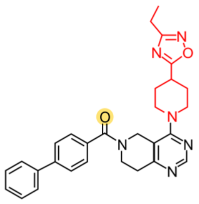	Hydrogen of the amine 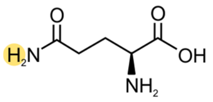	O-H 1.83
29	22	Q126 (Subunit B)	Hydrogen bond	Pyrimidine nitrogen	Hydrogen of the amine	N-H 1.95

				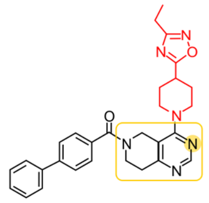	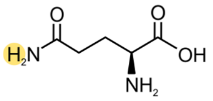	
30	22	G236 (Subunit A)	Polar	Substituent (C28 atom) 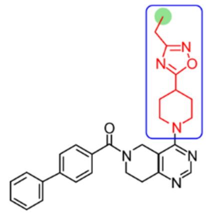	Hydrogen of alpha carbon 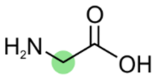	C-H 3.44
31	22	L239 (Subunit A)	Hydrophobic	Substituent (C23 atom) 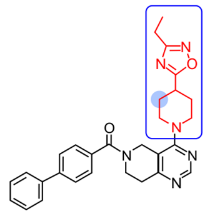	Delta methyl 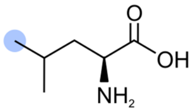	C-C 3.77
32	23	L244 (Subunit A)	Hydrophobic	Biphenyl (C16 atom) 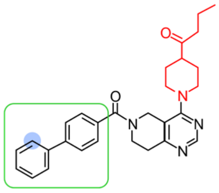	Delta methyl 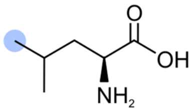	C-C 3.80
33	23	L244 (Subunit B)	Hydrophobic	Biphenyl (C18 atom) 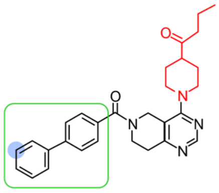	Delta methyl 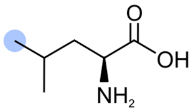	C-C 3.57
34	23	L247 (Subunit B)	Hydrophobic	Biphenyl (C11 atom) 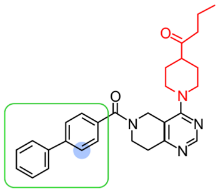	Delta methyl 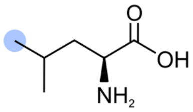	C-C 3.71
35	23	Q126 (Subunit A)	Hydrogen bond	Carbonyl	Hydrogen of the amine	O-H 2.59

				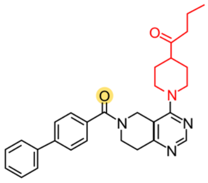	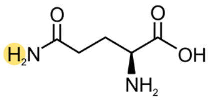	
36	23	L122 (Subunit A)	Hydrophobic	Pyrimidine carbon 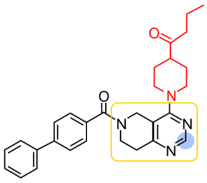	Delta methyl 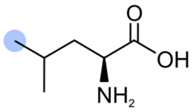	C-C 3.79
37	23	L239 (Subunit B)	Hydrophobic	Pyrimidine nitrogen 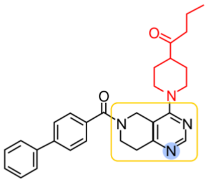	Beta carbon 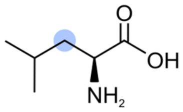	N-C 3.59
38	23	G236 (Subunit B)	Polar	Substituent (H of C27 atom) 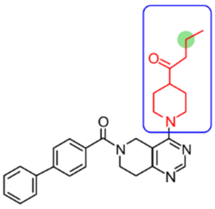	Alpha carbon 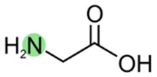	H-N 3.03

Interactions are presented in three different parts (blue, yellow and green squares) of the ligands, according to Fig 1A. Hydrogen bonds, hydrophobic and polar interactions are represented in yellow, blue and green spheres, respectively.

Table S5. Clusters of PK-THPP poses.

T3twiOO		T3tre2OO		T3tre1CC	
No. Cluster	Pop.	No. Cluster	Pop.	No. Cluster	Pop.
1	1	19	1	27	1
2	1	20	1	28	25
3	1	21	5	29	9
4	2	22	31	30	10
5	1	23	59	31	2
6	1	24	1	32	1
7	12	25	1	33	26
8	4	26	1	34	1
9	5			35	7
10	54			36	5
11	7			37	8
12	1			38	2
13	1			39	1
14	2			40	2
15	1				
16	4				
17	1				
18	1				
Pop. ave. =	5.556	Pop. ave. =	12.5	Pop. ave. =	7.143
SD =	12.434	SD =	21.454	SD =	8.42

Pop.: Population; **Pop. ave.:** Population average; **SD:** Standard deviation

Significant conformational clusters, for which the populations depart by more than 2*SD from the Pop. ave. are highlight in gray.

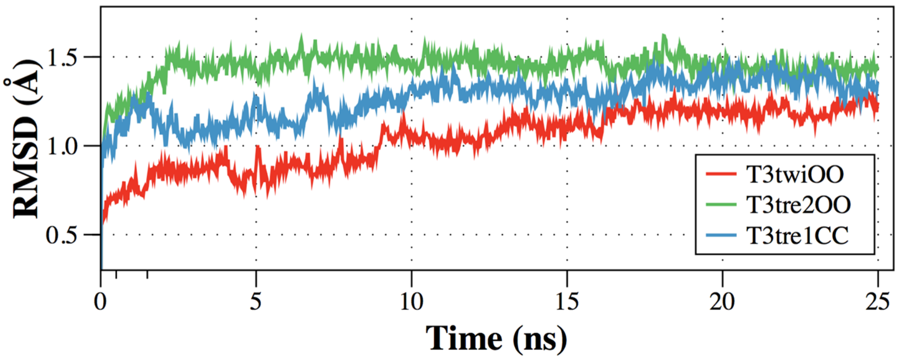

Figure S1. Time dependence of the RMSD backbone of TASK-3 models during 25ns MDs.

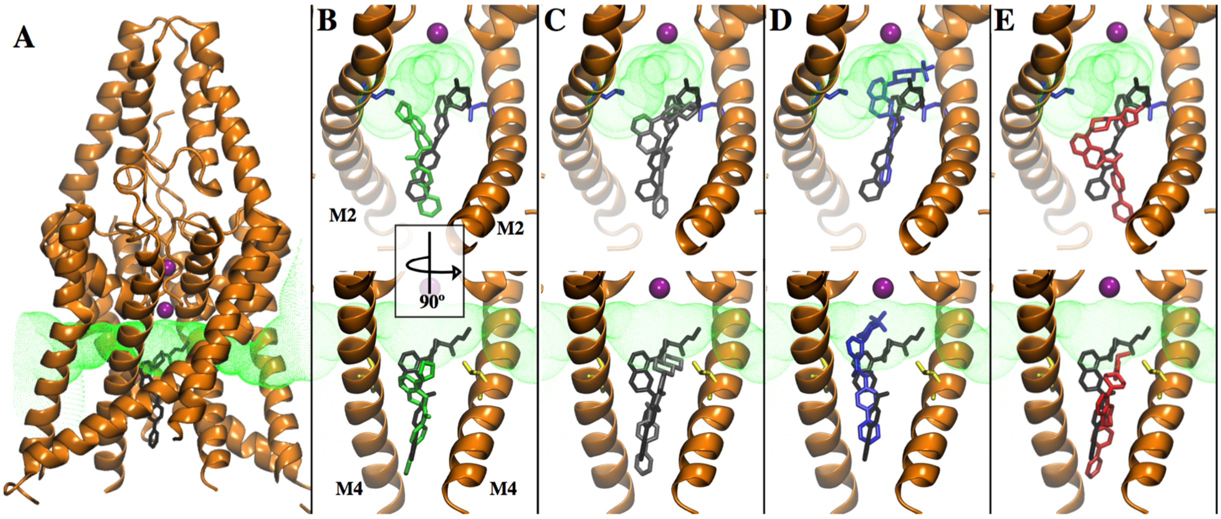

Figure S2. Binding of THPP derived compounds in T3tre2OO model. A. PK-THPP (black) is shown interacting at the interface between the fenestrations and the central cavity. B to E show the other compounds of the THPP-series in comparison with PK-THPP pose and representing the residues L122 (blue, upper panels) and L239 (yellow, downside panels). B. 17b (green), C. 20b (gray), D. 21 (blue), E. 22 (red). Compounds PK-THPP, 21, 20b and 22 reach the upper side of the fenestrations-inner cavity (green dotted surface) with a substituted piperidine group but not the compound 17b. For better representation TM1 and TM3 are not shown from figure B to G.

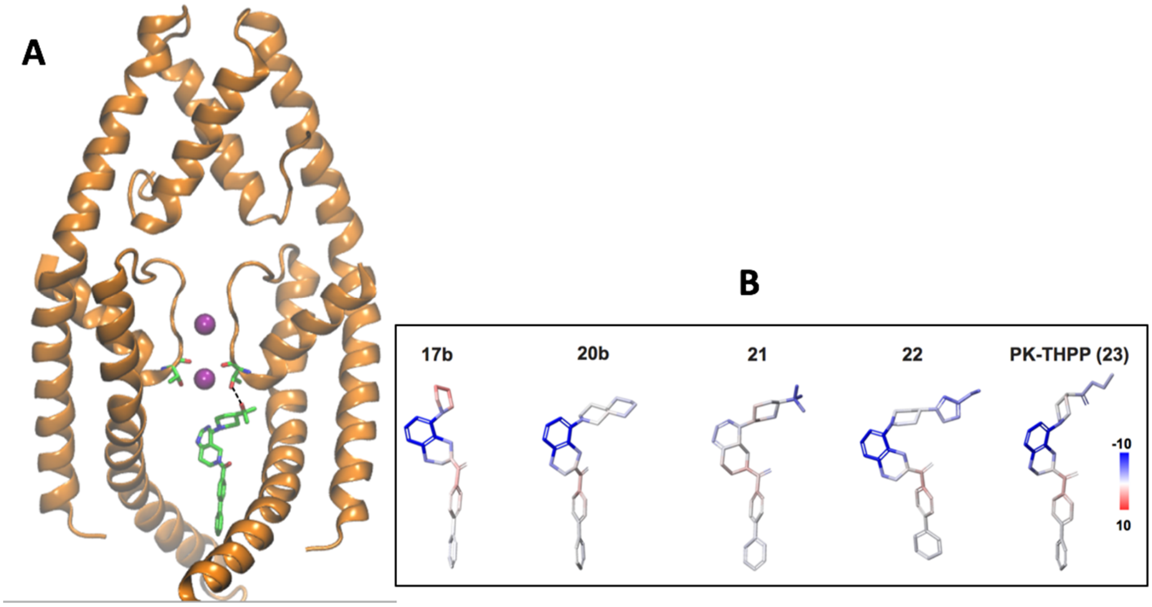

Figure S3. Compounds of THPP series with high affinity could establish a hydrogen bond with the threonines of the selectivity filter. A. From the selected docking poses of the THPP analogues, compound 21 establishes a hydrogen bond through the *A* group of the pharmacophore with T93 (represented in licorice). For better representation TM3 and TM4 are not shown. The distance between the oxygen of the *A* group of PK-THPP and T93 gamma oxygen is 2.8 Å. **B.** Relative ΔG_{Bind} values distribution along the molecules (in kcal/mol).

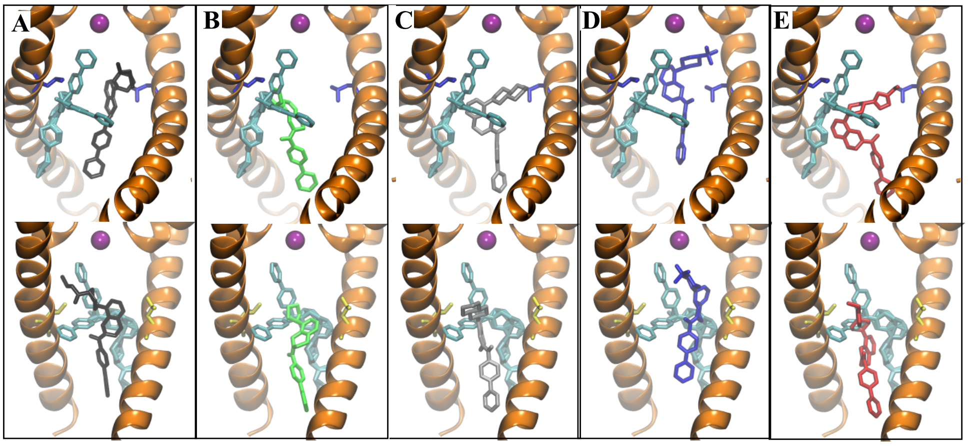

Figure S4. Binding of THPP derived compounds in TASK-3 in comparison with the THPP poses reported by Chokshi *et al.* [2] which are shown in cyan. Panels **A** to **E** show the compounds of the THPP-series in comparison with Chokshi *et al.* [2] PK-THPP poses; representing the residues L122 (blue, upper panels) and L239 (yellow, downside panels). **A.** The PK-THPP pose suggested by our SAR study (black) **B.** 17b (green), **C.** 20b (gray), **D.** 21 (blue), **E.** 22 (red) For better representation only TM2 is shown in the upper panels and TM4 in the downside panels.

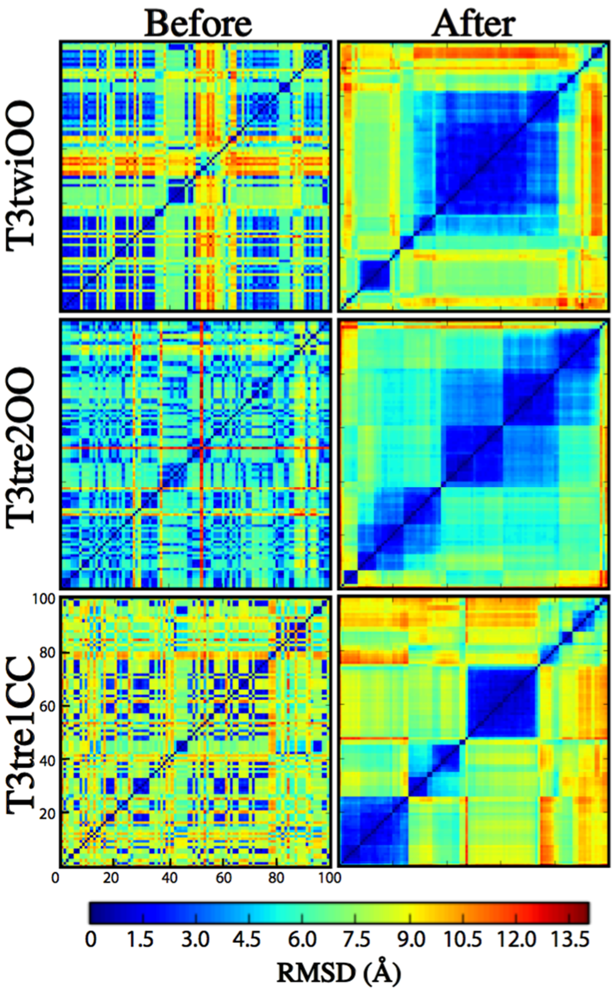

Figure S5. Ordering of PK-THPP docking poses in TASK-3 by cluster analysis. The symmetrical distance matrix illustrates atomic RMSD comparison of the 100 poses of PK-THPP found by molecular docking *per* model. On the diagonal line the atomic RMSD is zero because the poses are compared with themselves. Matrix of PK-THPP poses organized by number before and after clustering. Significant clusters are visible as squares on the diagonal. The inferior bar is the RMSD atomic distance scale in Å. Supplemental Table S4 shows all the clusters of PK-THPP poses *per* model, the mean cluster population, and the associated standard deviation (SD).

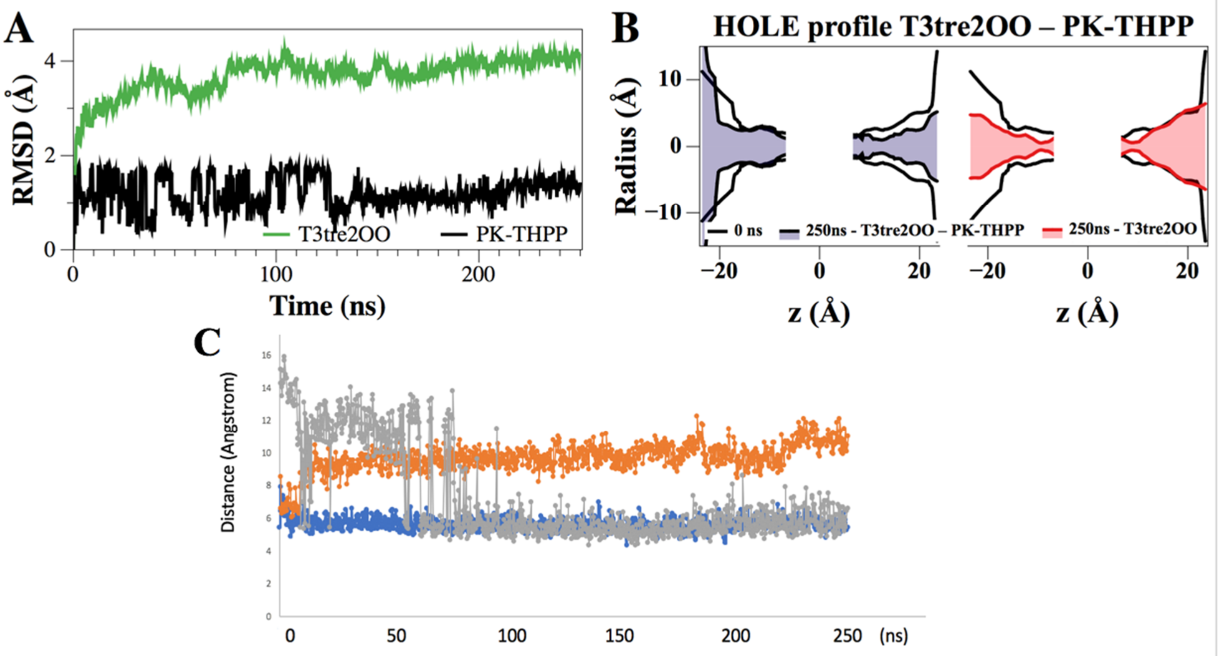

Figure S6. Analysis of the MDs of PK-THPP in T3tre2OO homology model. A. Time dependence of the RMSD for PK-THPP heavy atoms (black) and TASK-3 backbone atoms (green) during the 250 ns unrestrained MDs. **B.** *Left*, hole profile before (white) and after (purple) 250 ns MD simulations in the presence of PK-THPP, which prevents the movement to the 'up' state mainly in the left fenestration. *Right*, same analysis but without PK-THPP, revealing a closure of the side fenestrations (red) when the channels move to the 'up' state. **C.** Distances between the key actors in the left fenestration opening in the presence of PK-THPP during MDs. *As control*, the distance between the beta carbon of residue I118 (chain B) and the gamma carbon of L239 (chain A) without PK-THPP in the binding site is shown in blue. The distance between both atoms when PK-THPP is anchored at the binding site is in orange. The distance between carbon 35 of PK-THPP and the beta carbon of I118 (chain B) is shown in gray.

Supplemental references

1. Coburn, C. a; Luo, Y.; Cui, M.; Wang, J.; Soll, R.; Dong, J.; Hu, B.; Lyon, M. a; Santarelli, V. P.; Kraus, R. L.; Gregan, Y.; Wang, Y.; Fox, S. V; Binns, J.; Doran, S. M.; Reiss, D. R.; Tannenbaum, P. L.; Gotter, A. L.; Meinke, P. T.; Renger, J. J. Discovery of a pharmacologically active antagonist of the two-pore domain potassium channel K2P9.1 (TASK-3). *ChemMedChem* **2012**, *7*, 123–33, doi:10.1002/cmdc.201100351.
2. Chokshi, R. H.; Larsen, A. T.; Bhayana, B.; Cotten, J. F. Breathing Stimulant Compounds Inhibit TASK-3 Potassium Channel Function Likely by Binding at a Common Site in the Channel Pore. *Mol. Pharmacol.* **2015**, *88*, 926–934.
